# A Fault Tolerance Mechanism for On-Road Sensor Networks

**DOI:** 10.3390/s16122059

**Published:** 2016-12-03

**Authors:** Lei Feng, Shaoyong Guo, Jialu Sun, Peng Yu, Wenjing Li

**Affiliations:** State Key Laboratory of Networking and Switching Technology, Beijing University of Post and Telecommunication, Beijing 100876, China; syguo@bupt.edu.cn (S.G.); sunjialu@bupt.edu.cn (J.S.); yupeng@bupt.edu.cn (P.Y.); wjli@bupt.edu.cn (W.L.)

**Keywords:** on-road sensor network, fault tolerance, backup sensor deployment, route adjustment, cluster adjustment

## Abstract

On-Road Sensor Networks (ORSNs) play an important role in capturing traffic flow data for predicting short-term traffic patterns, driving assistance and self-driving vehicles. However, this kind of network is prone to large-scale communication failure if a few sensors physically fail. In this paper, to ensure that the network works normally, an effective fault-tolerance mechanism for ORSNs which mainly consists of backup on-road sensor deployment, redundant cluster head deployment and an adaptive failure detection and recovery method is proposed. Firstly, based on the *N* − *x* principle and the sensors’ failure rate, this paper formulates the backup sensor deployment problem in the form of a two-objective optimization, which explains the trade-off between the cost and fault resumption. In consideration of improving the network resilience further, this paper introduces a redundant cluster head deployment model according to the coverage constraint. Then a common solving method combining integer-continuing and sequential quadratic programming is explored to determine the optimal location of these two deployment problems. Moreover, an Adaptive Detection and Resume (ADR) protocol is deigned to recover the system communication through route and cluster adjustment if there is a backup on-road sensor mismatch. The final experiments show that our proposed mechanism can achieve an average 90% recovery rate and reduce the average number of failed sensors at most by 35.7%.

## 1. Introduction

**Figure 1:**
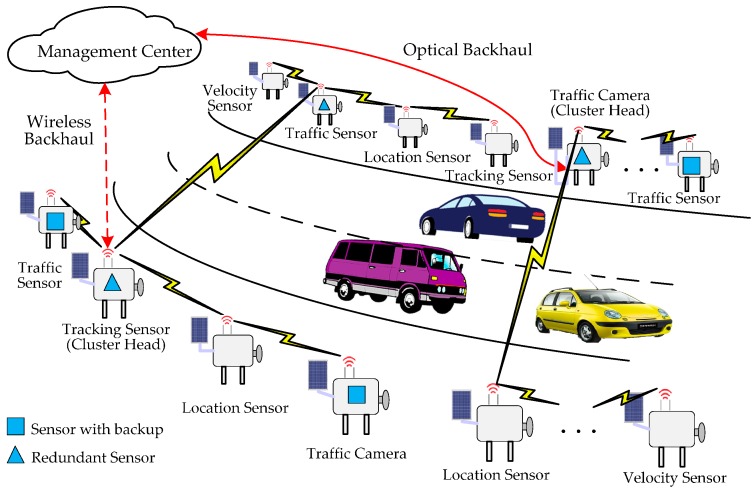
An illustration of an ORSN scenario.

With the rapid development of Intelligent Traffic Systems (ITSs), more powerful functions are required to observe traffic flow [1,2], track automobiles [3], signal incidents [4], measure gas pollution [5] and even ensure driving safety [6], etc. These functions are now supported by deploying more and more on-road sensors and sensors deployed along roads. For cost efficiency and flexibility, it is rarely practical to equip all these on-road sensors with cables or fibers, so wireless sensors enjoy a boost in type and quantity. Therefore, a typical On-Road Sensor Network (ORSN) is formed by a majority of wireless sensors communicating through radio links and certain sensors serving as data collectors and relaying collected data to a remote data center through radio or cable/fiber [7]. It boasts properties like flexible and easy deployment, two-way communication, and is distinguished from regular wireless sensor networks by its linear-like topology [8]. As illustrated in Figure 1, a typical ORSN scenario consists of a large number and variety of sensors for different ITS functions; however, as they are serving a common ITS system, they can cooperate and perform multi-hop data transmission to reduce energy consumption at individual sensor nodes [9]. To balance the energy consumption and traffic load/delay at intermediate nodes, it is preferred for these on-road sensors to form clusters and perform cluster-based management.

All this is a promising way to mitigate the increasingly severe traffic congestion and air pollution through ITS in urban areas, so lots of researchers are working on the functional aspects of ORSNs to support better traffic management [4]. Therefore, ITS is becoming a critical infrastructure to facilitate urban traffic, and its potential applications with high robustness requirements emerge, like ensuring the safety of automobiles and automatic vehicles. These direct our attention to the robust and fault-tolerance characteristics of ORSNs. In this paper a fault tolerant wireless sensor network is defined to ensure the ORSN system will not be affected when one or more sensor nodes fail. This can be achieved by deploying some redundant sensors as backup nodes, but considering the cost, deploying backup nodes for each on-road sensor is not realistic, so our problem becomes how to deploy the redundant nodes for the on-road sensor system and how to implement self-healing in this system.

To solve this problem, three aspects need to be considered: (1) how to deploy backup on-road sensors and consider communication reliability within a cost constraint? (2) how to deploy some redundant cluster heads to improve the network resilience? and (3) when the fault occurs, how to realize adaptive failure detection and recover the communication? In response to these questions, a fault tolerant architecture for an on-road sensor network is explored, and based on this architecture, a fault tolerant mechanism is proposed. The main contributions of this paper include:

1. A fault tolerant architecture for an on-road sensor network is proposed.
2. Two optimization models of how to deploy the backup sensors and the redundant cluster heads are proposed.
3. An algorithm to solve these deployment optimization models is proposed.
4. A protocol of how to adaptively detect and recover the faults in the on-road sensor system is proposed.

The remainder of the paper is organized as follows: Section 2 analyzes the related work. In Section 3, the architecture of the on-road sensor network and a design method for its fault tolerance are proposed. In this paper, the problems are converted to how to deploy the backup or redundant nodes for an on-road sensor network and how to implement system self-healing. The optimization model for the problems, the detailed constraint analysis, and the corresponding solving algorithm are given in Section 4. Section 5 discusses the simulation results. Finally, Section 6 concludes the paper.

## 2. Related Work

Many existing works are concerned with the effective and economical deployment of on-road sensors for better traffic observation or measuring performance. Among these works, Kianfar et al. [7] proposed optimizing traffic sensor locations for freeway bottleneck identification. Gupta et al. [10] stated there is a minimum number of sensors required for the desired level of coverage and connectivity, assuming sensors are stochastically deployed along the roadsides. Leyre et al. [11] utilized deterministic tools and approaches to analyze radio propagation in emulating scenarios of complex realistic traffic intersections to determine the best locations to deploy traffic sensors. Fernández-Lozano et al. [12] proposed a system based on Wireless Sensor Network (WSN) designed to characterize urban traffic, particularly traffic trend monitoring through the calculation of the origin-destination matrix. Masek et al. [13] outlined a perspective on a novel modular environment for traffic modeling, which allows recreating the examined road networks in their full resemblance. These studies focus merely on providing more accurate observations or measurement results from a well-established wireless sensor network. However, as ORSNs are evolving towards infrastructure to support driving safety [6,14], more requirements are being placed on ORSNs, especially on their robustness and fault-tolerance.

Due to the instability of wireless communication channels and the common fault-prone properties of all IT devices, the fault-tolerance of a wireless sensor network is of general concern in various fields. The schemes in these researches vary greatly, but basically within the ranges of fault-tolerant network physical topology construction and resilient network function recovery. The former elaborates on WSN construction considering robustness requirements. Among relevant existing works, Bhuiyan et al. [15] proposed to deploy backup sensors to guarantee a specified degree of fault tolerance in WSN-based structural health monitoring systems. Li et al. [16] considered the probabilistic sensor failures and developed a reliable traffic surveillance sensor location model to optimize the expected traffic surveillance benefits. The latter focuses on developing approaches to ensure the normal function of an established WSN after a node failure occurs. Within this scope, Korkali et al. [17] discussed an optimal sensor deployment procedure to ensure the robust and unique location of line faults appearing in smart grids by proposing fault-tolerant data processing algorithms to identify data from malfunctioning sensor nodes. Roumane et al. [18] improved conventional routing to better stabilize the throughput, reduce the average jitter, provide low control overhead and decrease the energy consumption of networks by introducing the second hop information in the route construction phase. Rico and Aboobeker et al. [19,20] proposed cluster head rotation-based mechanisms to avoid fast power supply failure of energy constrained wireless sensor networks. In addition, stability is critical in some industrial systems, so *N* − *x* becomes a general principle [21], which requires the system retain functionality even in the event of *x* component failures. Considering this principle, Shi et al. [22] mainly addressed the better trade-off between redundancy-based reliability ensurance and its cost by developing a quantitative analysis model. Wang et al. [23] proposed to improve the fault tolerance of WSNs for power line surveillance by both deploying redundant relay nodes and developing a self-adaptive fault modification algorithm for faulty paradigms based on minimizing expected component failure risks.

The above works all concern robustness and fault-tolerance of wireless sensor networks, but, to the best of our knowledge, research on fault tolerance dedicated to ORSNs is very limited. This is partially due to the low robustness requirements for ORSNs for traffic surveillance or observations, and on the other hand, it is subject to development of ITS where not many sensors had to be controlled or managed in the system before. However, ITS is playing more and more important roles in the traffic management, and its potential applications with high robustness requirements emerge, especially for safe driving and automatic vehicles.

Hence, in this paper, based on the characteristic of ORSNs, a fault tolerance framework is established to deliver a more comprehensive and in-depth solution to address this problem, which involves different stages including planning, deployment, operation and evaluation of the network. Specifically, firstly we give the backup on-road sensor deployment method with a consideration of cost and *N* − *x* role to guarantee the communication reliability. Then a redundant cluster head deployment model is introduced to improve the network resilience. Finally, an adaptive detection and resumption protocol is designed to recover from the communication problems caused by sensors’ faults.

## 3. System Architecture

In this section, the architecture of an on-road sensor system is presented first, and then a design method for a fault tolerant on-road sensor system is given. Finally, put forward three problems and give the solutions in this paper.

### 3.1. Architecture of the On-Road Sensor Network

Considering the scenario of freeways or bridges, on-road sensors which are used to collect and convey traffic information generally should be deployed in a symmetrical position on either side of the highway or bridge. The management center, which is usually deployed in a city or town, is a management entity to manage sensors and analyze the collected traffic information by neural network techniques [8]. Therefor all the on-road sensors need to communicate with the management center to transfer information or obtain some signals.

**Figure 2:**
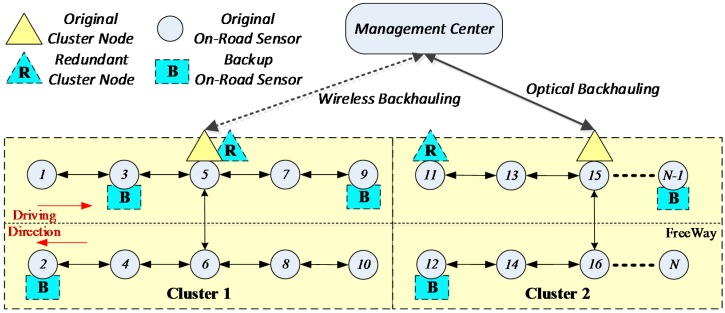
Architecture of an On-Road Sensor Network.

Generally, the management center is far away from the on-road sensors, so it is necessary to deploy a node having long-distance transmission capabilities for the on-road sensors to communicate with the management center. The long-distance communication can be provided by optical fiber or wireless backhauling (4G or future 5G). However, if each on-road sensor must communicate with the management center directly, the cost is very high, so the long-distance communication nodes can only be deployed on limited sensors. Hence several sensors form a cluster and the long-distance communication node is called the cluster head. In this scenario, most sensors exchange information with the cluster heads via a low-cost hop-by-hop short-distance communication technology (such as Zigbee). Then these cluster heads exchange information with the management center via cellular or optical fibre communication. Therefore, an ORSN includes three kinds of nodes: sensor nodes, cluster heads and the management center. The hybrid network architecture presented by this paper is shown in Figure 2.

As shown in Figure 2, the circles represent the on-road sensor nodes. In order to make sure the system can work normally when one or some of the sensors fail, some backup on-road sensors are needed. The shadowed box marked with **B** in Figure 2 represents the backup on-road sensor. Triangles represents the cluster heads which communicate with the management center through some long-distance communication technology, and the shadowed triangle marked with **R** represents a redundant cluster head which can form a denser on-road sensor system or play a backup role for the original cluster heads. Because the cost of cluster heads is high, the number deployed is limited. The on-road sensors without cluster heads need to transfer information to cluster heads by a hop-by-hop manner using some short-distance communication technology, and then communicate with the management center via the cluster head.

The on-road sensors need to transmit many kinds of traffic information to the management center and receive control signals from the management center along the opposite path, so the communication is two-way. The topology of the on-road sensor network is linear, while the topology of on-road sensors with cluster heads and the management center is star style. In this paper, we assume that all the on-road sensors can be synchronized by the management center and have no security problems. Hence, we mainly focus on improving the wireless communication reliability by a fault-tolerant mechanism, which concerns whether the on-road sensors can normally communicate with the management center in this topology.

According to the actual deployment of on-road sensors along the highway, given an undirected graph ***G*** = (***V***, ***E***), where ***V*** represents the on-road sensors, ***E*** represents the communication relationship among on-road sensors and then *N* = |***V***|.

Because sensor nodes communicate with the management center through cluster heads in a hop-by-hop style, naturally, the sensor nodes that communicate with the management center through the same cluster head form a cluster. In general, the on-road sensors are deployed with the cluster head located in the center of a cluster (like nodes 5 and 15 shown in Figure 2), which allows the on-road sensors on both sides to communicate with the cluster head over the shortest distance.

Here, the coverage of the cluster is discussed. Assuming that the maximum distance between any two adjacent sensor nodes in the *k*-th cluster is $\underset{\forall m,n}{\max}\left|v_{m}-v_{n}\right|$ $\underset{\forall m,n}{\max}\left|v_{m}-v_{n}\right|$, and the best coverage of the cluster head within the cluster is $R_{k}$, then the number of sensor nodes $C_{k}$ that can be covered by the *k*-th cluster head is expressed using Equation (1):

(1) $$C_{k}=4\times \frac{R_{k}}{\underset{\forall i,j}{\max}\left|v_{i}-v_{j}\right|}+2$$

If the coverage of two adjacent cluster heads overlaps, then the closer one will be chosen as the relay node. As shown in Figure 2, cluster 1 includes sensor nodes 1~10 within the rectangle area, and the cluster head is deployed on node 5, which is a relay node. We take node 10 as an example. Node 10 transfers information through {10-8-6-5} to node 5 (relay node), and then interacts with the management center through node 5. At the same time, there are backup sensors deployed on nodes 1, 4, 9, and a backup cluster head deployed on node 5 in cluster 1.

### 3.2. Tolerance Framework

In addition to the characteristics of two-way communication and hybrid network topology mentioned above, the ORSN system also need to satisfy other characteristics, such as high reliability and low cost, etc. The system should meet the requirement of the *N* − *x* principle, which means that for a system with *N* nodes, when *x* nodes are faulty, the system will not be affected. The *N* − *x* principle is widely applied when planning and design high reliable communication systems [21,22,23]. In this paper, we introduce this principle to the on-road sensor communication system in order to improve the reliability.

**Figure 3:**
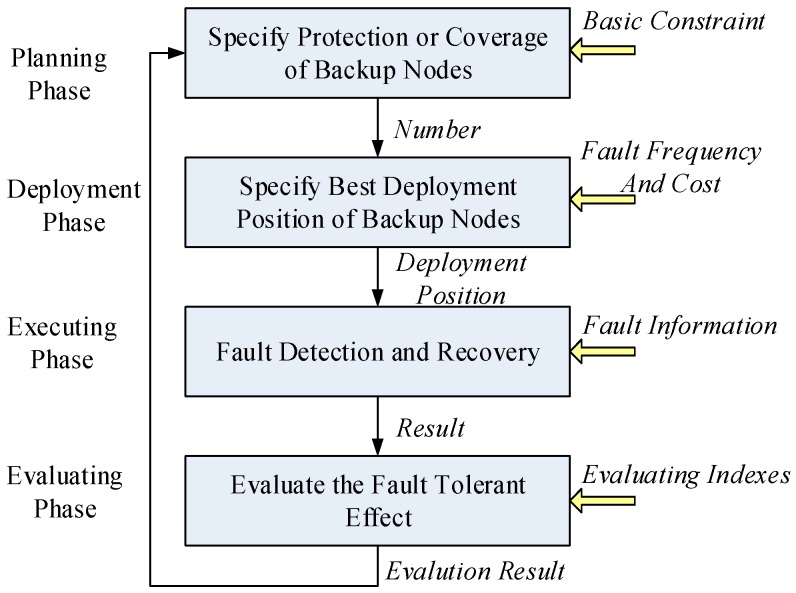
The design proposal of a fault tolerant On-Road Sensor Network.

Under the guidance of these requirements, to design a fault tolerant on-road sensor system, four phases should be considered: planning, deployment, operation, and evaluation, as shown in Figure 3. In the planning phase, the minimum required number of backup sensor nodes and redundant cluster heads will be given. In the process of determining the amount, some basic constraints, such as the *N* − *x* principle and the cluster coverage should be considered. In the deployment phase, the deployment positions of backup sensors and redundant cluster heads will be calculated based on the cost and failure rates of different on-road sensors. In the execution phase, the adaptive fault detection and recovery mechanism will be designed. When some on-road sensors or some cluster heads are faulty, the failure nodes should be automatically replaced, and when necessary the hop-by-hop routes need be re-planned or the clusters need to be reorganized, so as to achieve the purpose that the network services shall not be affected. In the evaluation phase, the running quality of the ORSN will be investigated according to the evaluation indexes, and the evaluation results will be input into the planning phase, as one of the inputs for ORSN planning.

So far, this paper has proposed the architecture of the ORSN and the design ideas for a fault tolerance system. Subsequent sections will discuss these phases in detail, put forward the problem model and give the solving method.

## 4. Modeling and Problem Solving for Best Backup Sensor Deployment and Adaptive Fault Recovery

When designing a fault tolerant ORSN, we consider factors such as reliability, protection, cost, coverage of the cluster and the network scalability, etc. In this paper, we assume that the sensor node and cluster head faults are independent of each other.

As a consequence of the separation between backup sensor node deployment and redundant cluster head deployment, the design of a fault tolerant ORSN is equivalent to three sub-problems: *Best Backup Sensors Deployment Problem*, *Best Redundant Cluster Heads Deployment Problem* and *Adaptive Fault Detection and Recovery problem*. The first problem is how to find the optimal deployment positions for the backup sensors according to the locations and failure rates of the on-road sensors, the cost, and the minimum number of backup sensors required according to the *N* − *x* principle. The second problem is how to design the optimal deployment positions for the redundant cluster heads. This problem considers factors such as the deployment position’s significance, the cost and the coverage of the cluster. The third problem is how to detect faulty nodes and how to adaptively re-plan the routes or reorganize the cluster when it is necessary to make sure that all the traffic information can be exchanged normally. These three sub-problems will be analyzed in details and the solutions will be given in the following sections. The main notations used in this paper are shown in Table 1.

### 4.1. Best Backup Sensors Deployment Problem

#### 4.1.1. Problem Model

The most reliable protection method is to deploy backup sensors in each position where a sensor can fail, but this approach is not feasible when considering the cost, so the deployment of backup nodes is selective. The deployment position and the number of backup sensors should be determined based on the *N* − *x* principle, cost and the original sensor’s importance.

The significance of each original sensor is designed by considering the requirement of sensor’s failure tolerance. Because the communication reliability is determined by whether this node can normally communicate with the cluster head through hop-by-hop transmission, the higher the failure rate of the sensor is, the higher the probability of the sensor losing its communication with cluster heads, hence this kind of on-road sensors have a higher deployment significance. This paper expects to obtain a maximal sum of sensor’s deployment significance, which is in contrast with the consideration of low deployment cost. Therefore, this optimization problem can be defined as Equation (2):

(2) $$\begin{array}{l} \max\left[\sum_{i}significance_{i}\left(backup\_sensors\right)\right]\\ \min\left[\sum_{i}\cos t_{i}\left(backup\_sensors\right)\right] \end{array}$$

which is subjected to the constraint of the *N* − *x* principle.

The above model presents a best deployment problem of backup on-road sensors that allows a balance between the cost and significance which represents the ability of fault tolerance when considering the *N* − *x* principle. We discuss this more specifically in the next section.

#### 4.1.2. Analysis

1. Backup Sensor Nodes Cost Modeling

The cost of backup sensors is determined by the equipment purchase cost and deployment engineering. In addition, considering the network scalability, the reserved cost for network expansion should be considered. Given the vector $SP=\{SP_{i},\;i=1,2,\ldots N\}$, where $SP_{i}$ represents whether there is a backup sensor node for the *i*-th original on-road sensor. $SP_{i}$ is a (0, 1) binary variable. Hence the minimum cost deployment optimization model can be defined as follows:

(3) $$\min\left[\left(1+RR\right)\cdot E_{s}\cdot N_{s}\right]$$

where the number of backup on-road sensors $N_{S}=\sum SP_{i}$. It should be noticed that $(1+RR)\cdot E_{s}$ is an assumed cost factor constant, which is determined by the procurement and deployment expense $E_{s}$ and the network scalability coefficient RR. Hence the above minimum cost model is equivalent to the minimum number of backup sensors problem: $\min:N_{S}=\sum SP_{i}$.

2. *N* − *x* Constraint

Assume that the on-road sensor faults are independent of each other, so the distribution probability of faults in *N* nodes obeys a Bernoulli distribution. At the same time, there is the probability that each fault is also different because of the different fault types. Based on the above assumption, the fault probability of the *i*-th node is noted as $p_{i}$, the probability of no fault case is noted as $q_{i}=1-p_{i}$, and the total fault sensor’s number is noted as *X*. The probability that *x* nodes fail among *N* nodes is noted as P{X=x}(x=1,2,3…,N). When *x* = 1, it means only one node fails in *N* nodes. In this case, the system should work normally according to the *N* − 1 principle. Likewise, P{X=x} means there are *x* failed nodes among *N* nodes, and in this case, the system should also work normally according to the *N* − *x* principle.

According to the nature of the Bernoulli distribution, we have:

(4) $$P\{X=x\}=\left(\begin{array}{c} N\\ x \end{array}\right)p^{x}q^{N-x}$$

Then we assume μ is the expectation of *X*. Since *X* follows a binomial distribution, according to Equation (4), μ can be obtained by:

(5) $$\mu =E\left[X\right]=\sum_{i=1}^{N}p_{i}$$

The upper bound of the binomial distribution is defined as:

(6) $$P\{X=x\}\leq {\left(\frac{\sum_{i=1}^{N}p_{i}}{x}\right)}^{x}{\left(\frac{\sum_{i=1}^{N}q_{i}}{N-x}\right)}^{n-x}={\left(\frac{\mu }{x}\right)}^{x}{\left(\frac{N-\mu }{N-x}\right)}^{N-x}$$

In order to fulfill the *N* − *x* principle, the number of backup on-road sensors must satisfy the following equation:

(7) $$\sum_{i=1}^{N}SP_{i}\geq N\cdot {\left(\frac{\mu }{x}\right)}^{x}{\left(\frac{N-\mu }{N-x}\right)}^{N-x}$$

**Figure 4:**
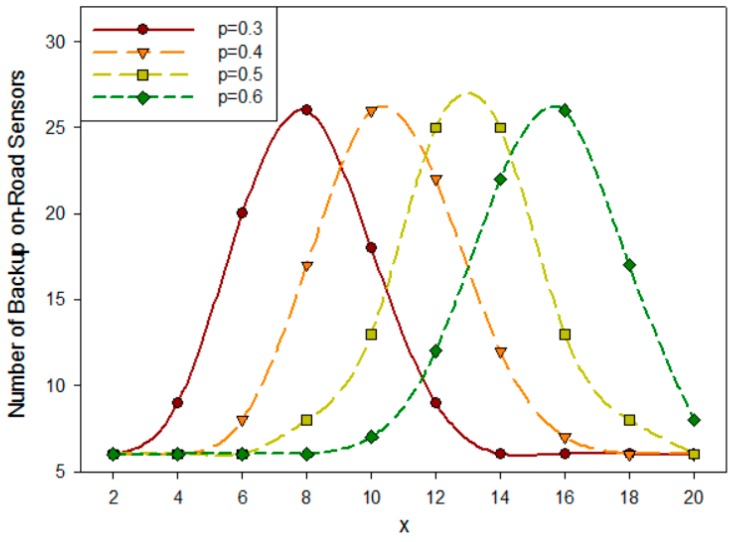
The relation among number of backup sensors, failure rate and *x*.

From Equation (7) we can know that, for a freeway of a certain length, the system’s failure rate and the required reliability (represented by *x* in the *N* − *x* principle) directly affect the number of backup on-road sensors. Figure 4 depicts the relation between the number of backup sensors and *x* in the *N* − *x* principle.

Based on the characteristics of the binomial distribution, it is symmetric around the point $x=\mu =\sum_{i=1}^{N}p_{i}$. To simplify our analysis, we will choose x≤μ in the following discussion. As a result, the number of backup sensor nodes increases with the growth of *x* in the *N* − *x* principle and the growth of fault probability *p*, causing deployment cost increases too.

3. Significant Weight

Assume that the probability of the *j*-th class fault of the *i*-th node during a period of time is $F_{ij}$ $F_{ij}$ expressed by Equation (8):

(8) $$F_{ij}=\frac{\delta_{ij}}{\delta_{total}}$$

where $\delta_{ij}$ represents the number of the *j*-th class faults of the *i*-th node during the statistical period, and $\delta_{total}$ represents the total number during the statistical period. For all the *M* class faults, the total fault probability of the *i*-th node is expressed by Equation (9):

(9) $$F_{i}=\sum_{j=1}^{M}F_{ij}$$

In order to ensure that the node with the higher fault frequency has the higher priority when deploying the backup on-road sensors, we set different weights for nodes according to their different failure rates. The higher the failure rate is, the higher the weight is. We define $\varphi_{L}$ and $\varphi_{H}$ as the low threshold and high threshold of the failure rate, respectively. Then the weight for the *i*-th node is expressed as $w_{i}$ which is calculated by Equation (10):

(10) $$w_{i}=\{\begin{array}{l} {\left(\frac{F_{i}}{\varphi_{H}-\varphi_{L}}\right)}^{2}{\;\mathrm{if}\;F}_{i}<\varphi_{L}\\ \frac{F_{i}-\varphi_{L}}{\varphi_{H}-\varphi_{L}}\;\mathrm{if}\;\varphi_{L}<F_{i}<\varphi_{H}\\ \frac{F_{i}}{\varphi_{H}-\varphi_{L}}\;\mathrm{else} \end{array}$$

The principle of the design on the weight $w_{i}$ is to scale the significance of the sensors with too high or too low failure rate, then we can give the maximum significant deployment objective function:

(11) $$\max\;W_{s}=\left[\sum_{i=1}^{N_{s}}w_{i}\cdot SP_{i}\right]$$

#### 4.1.3. Solution on Backup On-Road Sensors Deployment

Through the analysis above, then the *Best Backup Sensors Deployment Problem* can be converted into:

(12) $$\begin{array}{l} \max\;W_{s}=\left[\sum_{i=1}^{N}w_{i}\cdot SP_{i}\right]\\ \min\;N_{s}=\sum_{i=1}^{N}SP_{i}\\ s.t.\;N_{s}\geq N\cdot {\left(\frac{\mu }{x}\right)}^{x}{\left(\frac{N-\mu }{N-x}\right)}^{N-x} \end{array}$$

It can be seen that the maximum significance objective is contrary to the minimum cost objective. Hence, for this two-objective optimization problem, we use the ideal point method to construct a single objective optimization problem. We can easily know that the optimal solution $N_{s}^{*}$ on the separated minimum cost problem is $\left[N\cdot {\left(\frac{\mu }{x}\right)}^{x}{\left(\frac{N-\mu }{N-x}\right)}^{N-x}\right]+1$ according to the *N* − *x* principle constraint, where operator [⋅] represents the rounded down operation. The optimal solution $W_{s}^{*}$ on the separated maximum significance problem is $\sum_{i=1}^{N}w_{i}$. Therefore, the problem (12) is transformed into:

(13) $$\begin{array}{l} \min f_{s}\left(SP\right)=\lambda_{1}{\left(\frac{\sum_{i=1}^{N_{s}}SP_{i}-N_{s}^{*}}{N_{s}^{*}W_{s}^{*}}\right)}^{2}+\lambda_{2}{\left(\frac{\sum_{i=1}^{N_{s}}w_{i}\cdot SP_{i}-W_{s}^{*}}{N_{s}^{*}W_{s}^{*}}\right)}^{2}\\ s.t.\;g_{s}\left(SP\right)=N\cdot {\left(\frac{\mu }{x}\right)}^{x}{\left(\frac{N-\mu }{N-x}\right)}^{N-x}-\sum_{i=1}^{N_{s}}SP_{i}\leq 0 \end{array}$$

where $0\leq \lambda_{1}\leq 1$ and $0\leq \lambda_{2}\leq 1$ to define the objective fairness between the cost and significance. However, we still need to solve this 0–1 integer problem which has a quadratic term. This paper proposes an algorithm combined by the Sequential Quadratic Programming (SQP) and Branch-and-Bound (BB) algorithm to solve this problem. Firstly, we do the relaxation operation for converting the original problem (13) into one with the continuous variable $SP^{\prime }=\left\{SP_{i}^{\prime }\left|0\leq SP_{i}^{\prime }\leq 1\right|\right\}$. We consider the solution on SQP’s *k*-th iteration is $S{P^{\prime }}^{k}$. From (13) we can find out that $\nabla f_{s}\left(S{P^{\prime }}^{k}\right),\;\nabla^{2}f_{s}\left(S{P^{\prime }}^{k}\right),\;\nabla g_{s}\left(S{P^{\prime }}^{k}\right)$. Let $s^{k}=SP^{\prime }-S{P^{\prime }}^{k}$ then, we can construct a QP problem for (13) on the basis of a Taylor expansion:

(14) $$\begin{array}{l} \min f_{s}\left(SP\right)=\frac{1}{2}S^{k}\cdot \nabla^{2}f_{s}\left(S{P^{\prime }}^{k}\right)\cdot S^{k}-\nabla f_{s}{\left(S{P^{\prime }}^{k}\right)}^{T}\cdot S^{k}\\ s.t.\;\nabla g_{s}{\left(S{P^{\prime }}^{k}\right)}^{T}\cdot S^{k}+g_{s}\left(S{P^{\prime }}^{k}\right)\leq 0 \end{array}$$

Then the SQP method is used to solve the relaxed problem of (13), which is given in Algorithm 1.

**Algorithm 1.** SQP solving algorithm for the relaxed problem of (13).1: Initialization: the iteration k=0, the initial point $S{P^{\prime }}^{0}$ which makes $\nabla^{2}f(S{P^{\prime }}^{0})=I$ and the convergence precision ε. 2: At the *k*-th iteration with solution $S{P^{\prime }}^{k}$, covert the original relaxed problem into QP form as (14). 3: Work out (14) by Lagrange multiplier method and lets $s^{k}=s^{*}$. 4: To find $S{P^{\prime }}^{k+1}$ on the ray $S{P^{\prime }}^{k+1}=S{P^{\prime }}^{k}+\alpha^{k}S^{*}$ where $\alpha^{k}$ is the standard step size parameter 5: If $S{P^{\prime }}^{k+1}$ satisfies convergence precision ε, then the optimal solution $SP^{\prime }*=S{P^{\prime }}^{k+1}$; otherwise, next to step 6. 6. Modify $\nabla^{2}f(S{P^{\prime }}^{k+1})$ by BFGS formula. 7. Let k=k+1 and repeat to step 2.

After SQP solves the continuous relaxed problem, combined with the BB method, we can find the feasible integer solution of the original problem. The whole algorithm is given in Algorithm 2.

**Algorithm 2.** SQP-BB solving algorithm.1: Do continuous relaxation on problem (13) by replacing X∈{0,1} by $X\in {\left[0,1\right]}^{n}$. 2: Use SQP to find optimal solution for nonlinear programming problems (NLPs) on relaxed range. 3: If all variables in $SP^{\prime }$ are integer, end. Otherwise, do next. 4: *i-*th point to the first non-integer $SP_{i}^{\prime }$. 5: Branch on $SP_{i}^{\prime }$ and add $SP_{i}^{\prime }=0$ and $SP_{i}^{\prime }=1$ bounds respectively to the NLP relaxation. Solve two new NLP problems with SQP respectively and choose one solution with higher objective value. This will determine $SP_{i}^{\prime }=0$ or $SP_{i}^{\prime }=1$. 6: Update the optimal objective value and solution vector $SP^{\prime }$, repeat to 3.

The proposed algorithm in the above flow chart mainly consists of three steps. Step 1: We firstly relax problem (12) into one with a continuous 0–1 variable $SP^{\prime }$ and construct a normal QP problem in (14). SQP algorithm in Algorithm 1 is used to solve this relaxed problem and the optimal solution $S{P^{\prime }}^{*}$ is worked out; Step 2: Through brand-and-bound method, the non-integer term $SP_{i}^{\prime }$ in $S{P^{\prime }}^{*}$ is rounded into 0 and 1 respectively. It forms two branch sub-problems when these two conditions are added into the relaxed problem, respectively. Hence the upper bound and lower bound can be found and we can continue to search the optimal solution between them; Step 3: Repeat step 2 until all the terms in $S{P^{\prime }}^{*}$ are 0 or 1, then the optimal solution on the original problem can be found $SP^{*}=S{P^{\prime }}^{*}$.

### 4.2. Best Redundant Cluster Heads Deployment Problem

#### 4.2.1. Problem Model

Commonly, the cluster heads are more important than the normal on-road sensors because of their roles in the on-road sensor system, which means the cluster heads provide the communications between a cluster of on-road sensors and the management center. The deployment positions and the number of redundant cluster heads should be determined by the coverage constraint, cost and the new deployed cluster head’s importance. It should be noticed that the failure rate of the cluster heads is normally lower than the sensors. Therefore, we can design the new deployed cluster heads’ significance weight more according to the delay (hops) improving rather than only the cost and fault tolerance. Therefore, this optimization problem can be defined as Equation (15):

(15) $$\begin{array}{l} \max\left[\sum_{i}significance_{i}\left(backup\_clusterNodes\right)\right]\\ \min\left[\sum_{i}\cos t_{i}\left(backup\_clusterNodes\right)\right] \end{array}$$

which is subjected to the coverage of the cluster head constraint. We will analyze the objective and constraint in the next section.

#### 4.2.2. Analysis

1. Redundant Cluster Heads Cost Modeling

Being different from the cost model of backup sensors, there are two cases when deploying the redundant cluster heads: One is that a cluster head already exists at the on-road sensor’s position. In this case, the deployment cost can be reduced a lot because of the reuse of the associated equipment or environment of the existed cluster head such as installation site, power supply unit and so on. The deployment cost in this case is $E_{L}$; another one is the case of a new deployment, whose cost is represented by $E_{H}$. Assume $CP=\{CP_{i},\;i=1,2\cdots ,N\}$, where $CP_{i}$ represents whether there is a redundant cluster head in the *i*-th on-road sensor. $CP_{i}$ is also a (0, 1) binary variable. Hence the minimum cost deployment optimization model can be defined as follows:

(16) $$\min\left[\left(1+RR\right)\cdot \sum_{i}E_{c}\left(i\right)\cdot CP_{i}\right]$$

where $E_{c}(i)=E_{L}$ if a redundant cluster head has been deployed in the *i*-th cluster head in which the original cluster head deploys; otherwise $E_{c}(i)=E_{H}$.

2. Coverage Constraint

Next, we will analyze the coverage of the redundant cluster heads. As a consequence of the importance of the cluster heads when backhauling in the ORSN, we consider the extreme case where the redundant cluster heads can cover all the on-road sensors if all the original cluster heads are faulty. Assume that the coverage radius of the *k-*th cluster is a same constant $R_{k}=R$, then the number of on-road sensors covered within this cluster can be calculated according to Equation (1). Because all the sensor nodes must be covered by the redundant cluster heads, the coverage constraint condition is expressed by Equation (17) with the premise that the on-road sensor system is divided into $K=N_{c}$ clusters:

(17) $$\sum_{i=1}^{N}\left[4\cdot \frac{R}{\underset{\forall m,n}{\max}\left|v_{m}-v_{n}\right|}+2\right]\cdot CP_{i}\geq N$$

3. Significant Weight

Due to the importance of the cluster heads, we have assumed that the redundant cluster heads can cover all the sensors in the above analysis. Therefore, being different from the design for the backup sensors we can take into account quality of service (QoS) when designing the significant weight. This is to say that through the deployment of redundant cluster heads, we can form a denser on-road sensor system with a reduction on the sensor’s delay or hops to transfer their traffic information to the management center, which can improve the reliability and QoS. Assuming the *i*_1_-th and *i*_2_-th on-road sensors are two adjacent nodes in which the cluster heads are deployed and $i_{1}\leq i\leq i_{2}$, then the significant weight of the redundant cluster head’s deployed position can be defined as:

(18) $$\partial_{i}=\frac{1}{\left|v_{i}-v_{i_{1}}\right|-\left|v_{i}-v_{i_{2}}\right|+\eta }$$

where η is an assumed constant. It can be seen from Equation (18) that we are inclined to deploy the redundant cluster heads at the mid-position between two original adjacent cluster heads in order to form a denser on-road sensor system to improve the on-road sensors’ access hops. Then we can give the maximum significant deployment objective function:

(19) $$\max\;W_{c}=\sum_{i=1}^{N}\partial_{i}CP_{i}$$

#### 4.2.3. Solution on Redundant Cluster Heads Deployment

Through the above analysis, the *Best Redundant Cluster Heads Deployment Problem* can be expressed by:

(20) $$\begin{array}{l} \max\;W_{c}=\sum_{i=1}^{N}\partial_{i}CP_{i}\\ \min\;\sum_{i=1}^{N}E_{c}\left(i\right)CP_{i}\\ s.t.\;\sum_{i=1}^{N}\left[4\cdot \frac{R}{\underset{\forall m,n}{\max}\left|v_{m}-v_{n}\right|}+2\right]\cdot CP_{i}\geq N \end{array}$$

In this paper, we assume the coverage of each cluster is the same in order to facilitate the analysis. Like the backup sensor deployment work, we use the ideal point method to convert Equation (20) into a single objective optimization problem. First of all, for the separated minimum cost problem, the number of the minimum redundant cluster heads $N_{c}=\sum_{i=1}^{N}CP_{i}$ $N_{c}=\sum_{i=1}^{N}CP_{i}$ under the coverage constraint is $N_{c-\min}=\left[N/(4\cdot \left[\frac{R}{\underset{m,n\in N}{\max}\left|v_{m}-v_{n}\right|}\right]+2)+1\right]$. Considering the number of the original cluster heads $N_{oc}$, the optimal objective function value of the separated minimum cost problem is:

(21) $$fo=\{\begin{array}{c} E_{L}\cdot N_{oc}+E_{H}\cdot \left(N_{c-\min}-N_{oc}\right),\;if\;N_{oc}\leq N_{c-\min}\\ E_{L}\cdot N_{c-\min},\;if\;N_{oc}\geq N_{c-\min} \end{array}$$

It can be easily known that the optimal objective function value of the separated maximum significance problem is $W_{c}^{*}=\sum_{i=1}^{N}\partial_{i}$. Therefore, Equation (20) is transformed into:

(22) $$\begin{array}{l} \min f_{c}\left(CP\right)=\lambda_{1}{\left(\frac{\sum_{i=1}^{N_{c}}E_{c}\left(i\right)\cdot CP_{i}-f_{0}}{W_{c}^{*}f_{0}}\right)}^{2}+\lambda_{2}{\left(\frac{\sum_{i=1}^{N_{c}}\partial_{i}CP_{i}-W_{c}^{*}}{W_{c}^{*}f_{0}}\right)}^{2}\\ s.t.\;g_{c}\left(CP\right)=N-\sum_{i=1}^{N}\left[4\cdot \frac{R}{\underset{\forall m,n}{\max}\left|v_{m}-v_{n}\right|}+2\right]\cdot CP_{i}\leq 0 \end{array}$$

To be the same as the *Best Backup Sensors Deployment Problem* in Equation (14), we can use the similar algorithm proposed in Figure 3 to work out the optimal solution $CP^{*}$ to problem (22).

### 4.3. Adaptive Fault Detection and Recovery Problem

#### 4.3.1. Definition

The decisions of the deployment positions of the backup on-road sensors and redundant cluster heads are the first two steps to design a fault tolerant ORSN. Next, when one or some faults actually occur, how to detect the failure nodes and how to quickly recovery from the faults are the important tasks to guarantee a high system reliability, so it is necessary to design an efficient and autonomic fault detection and recovery mechanism to guarantee that the traffic information exchanges are not affected.

Assume the *k*-th cluster in the system is expressed as $C^{k}=\{v_{i}^{k},\;i\in N\}$, where $v_{i}^{k}$ represents the *i*-th on-road sensor in this cluster. Suppose that there are *S* on-road sensor nodes in the cluster, that means $\left|C^{k}\right|=S$. In each cluster, only one master cluster head is deployed, which is expressed as $v_{c}^{k}$. SP is the position vector for backup sensor nodes, and CP is the position vector for redundant cluster heads. On-road sensors transfer information to $v_{c}^{k}$ through the hop-by-hop style. If a sensor $v_{i}^{k}$ fails, then the others in the routes will be affected and cannot transfer information to the cluster head $v_{c}^{k}$. To guarantee the information transfer of the affected on-road sensors, the hop-by-hop route needs be adjusted, or the structure of clusters even needs to be adjusted. Thus the adaptive fault detection and recovery problem is converted into how to detect the fault(s) and how to dynamically adjust the hop-by-hop routes and clusters when the faults occur.

#### 4.3.2. Analysis

Dynamic route adjustment and cluster adjustment should be done by the management node. When the management node detects a fault, it should localize the failed sensors firstly, and then execute the adaptive route adjustment and cluster adjustment algorithm when necessary.

1. Fault Localization and Recovery

First of all, the failed on-road sensors should be localized. Because the ORSN has a linear-like topology, we adopt the direct analysis method to localize the fault(s). For example, cluster 1 in Figure 1 is expressed as $C^{1}=\{v_{i}^{1},\;i\in 1\ldots 10\}$, and a cluster head is deployed in the node $v_{5}^{1}$. All the other normal on-road sensors transfer information to $v_{5}^{1}$ in a hop-by-hop style. There are timeout devices configured in the management center. If the management center does not receive the information from sensor nodes after a timeout, the corresponding sensors will be put into the fault set ***FV***, and therefore we have $FV=\{fv_{i}^{k},\;i\in [1\ldots N],k\in [1\ldots K]\}$, where $fv_{i}^{k}$ represents the node whose information has not been received by the management center after a timeout.

Assume $v_{6}^{1}$, $v_{8}^{1}$ and $v_{10}^{1}$ are the nodes whose information has not been received by the management center, then we can deduce that the node $v_{6}^{1}$ failed. If there is a backup on-road sensor deployed in the node $v_{6}^{1}$, then the failed sensor will be replaced by the backup one directly, thus the adaptive fault recovery is completed, but if there is no backup sensor deployed at the location, the route adjustment or cluster adjustment should be executed according to the actual conditions.

2. Adaptive Route Adjustment

When a node fails and no backup node is deployed, the other affected nodes will first reselect a relay node in the same cluster with the premise that the coverage constraint and minimal hop number constraint are satisfied. As mentioned above, when $v_{6}^{1}$ fails, $v_{8}^{1}$ and $v_{10}^{1}$ cannot transfer information via $v_{6}^{1}$. At the same time, nodes $v_{7}^{1}$ and $v_{9}^{1}$ are working normally, then the route of $v_{8}^{1}$ can be converted from {8-6-5} to {8-7-5}, and the route of $v_{10}^{1}$ can be converted from {10-8-6-5} to {10-8-7-5} or {10-9-7-5}. Thus the route adjustment is completed.

3. Adaptive Cluster Adjustment

When a node fails without a deployed backup one and other affected nodes cannot be rerouted within the same cluster at the same time, in this case the cluster adjustment will be executed. The cluster adjustment is performed by taking the coverage and hop numbers as constraints. For example, when the nodes $v_{6}^{1}$ and $v_{7}^{1}$ in Figure 2 both fail and without backup nodes deployed, the nodes $v_{8}^{1}$, $v_{9}^{1}$ and $v_{10}^{1}$ cannot transfer data to a cluster head in cluster 1, then those nodes are called island sensors.

**Figure 5:**
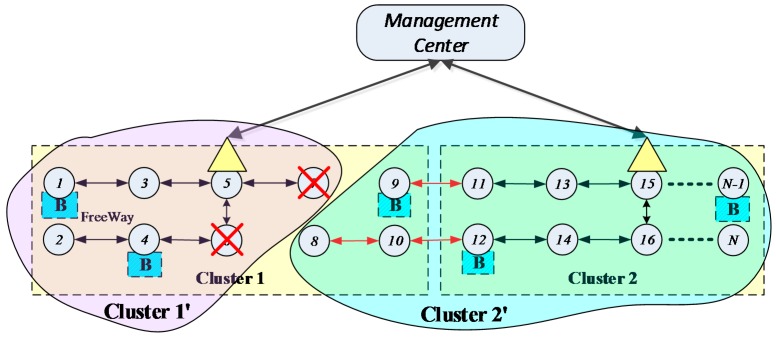
Illustration of cluster adjustment.

In this case, the island sensors, $v_{8}^{1}$, $v_{9}^{1}$ and $v_{10}^{1}$, can be adjusted to the adjacent cluster 2, until the number of island nodes is 0, thus the new clusters 1′ and 2′ are formed, as shown in Figure 5. We take the node $v_{10}^{1}$ as an example whose hop-by-hop route is adjusted to {10-12-14-16-15}, as shown in Figure 5. If the coverage in the cluster 2′ cannot cover one or some isolated nodes, and also no other cluster head can cover these nodes, these nodes will become actual island sensors. For example, if the cluster 2′ cannot cover the node $v_{8}^{1}$, then $v_{8}^{1}$ will become an actual island sensor. In this case, the backup nodes need be re-deployed and the corresponding information will be reported to the management center.

#### 4.3.3. Procedures of Adaptive Detection and Resume Mechanism

**Figure 6:**
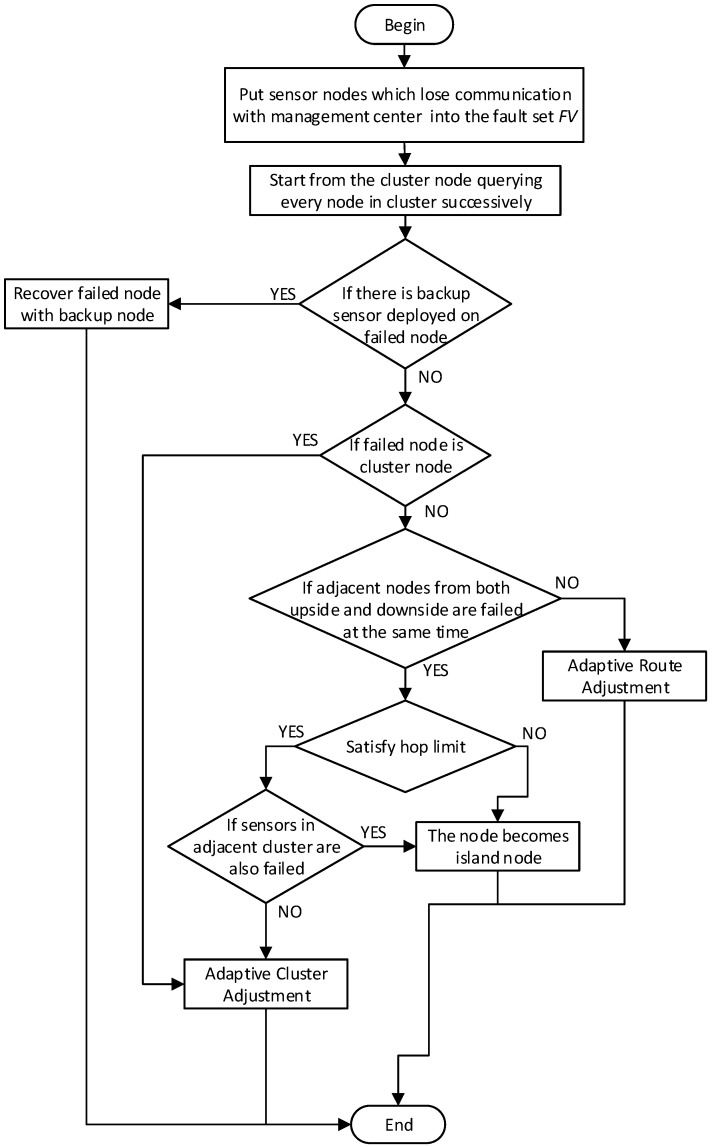
Flow of ADR mechanism.

The flow of the Adaptive Detection and Resume (ADR) protocol is as illustrated in Figure 6. The detailed procedures of dynamic recovery are given as follows: firstly, sensor nodes losing communication with management center will be put into the fault set which can be denoted by $FV=\{fv_{i}^{k},\;i\in [1\ldots N],k\in [1\ldots K]\}$. These failed sensors include two types: physically failed sensors without backup nodes deployed nearby, called directly failed sensors and normal sensor nodes losing communication because of other physically failed sensors, called indirectly failed sensors. This paper defines a detection window include four adjacent sensors from both upside and downside. From the beginning of the cluster head, we move the window step by step, querying the entire cluster. Adaptive route adjustment and cluster adjustment can be executed according to the following cases.

Case 1: Operate normally

If there is no failed sensor in the detection window, the window will move backwards directly.

Case 2: Adaptive Route Adjustment

**Figure 7:**
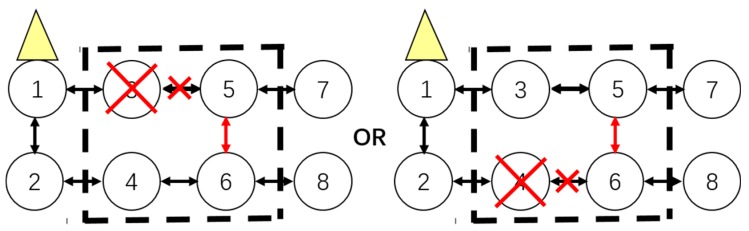
Situation of Route Adjustment.

If one of the sensors in the window which is on the upside or downside has failed, route adjustment should be executed as shown in Figure 7.

Case 3: Adaptive Cluster Adjustment

**Figure 8:**
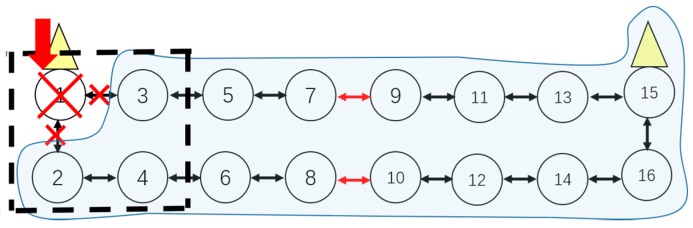
Cluster head failed.

**Figure 9:**
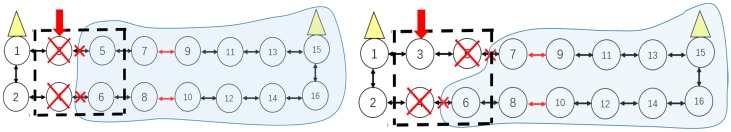
Situation of cluster adjustment.

(1) If a cluster head has physically failed, there should be a cluster adjustment, which is shown in Figure 8.
(2) If both sensor from upside and downside which are on the vertical or diagonal position in the window have failed at the same time, cluster adjustment should be executed as shown in Figure 9.

Case 4: Island Sensor

**Figure 10:**
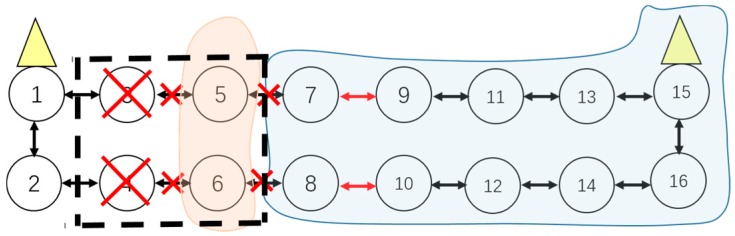
An island sensor situation.

**Figure 11:**
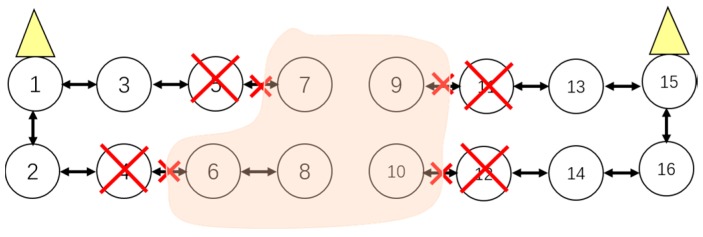
Another island sensor situation.

(1) Cluster adjustment should meet the hops constraint condition. The hops constraint limits the maximum number of sensors on one side of a cluster. Beyond the hops count limit, sensors will not be recovered resulting in a cluster of island sensors, which is shown in Figure 10.
(2) Figure 11 shows that if sensors which belong to adjacent cluster respectively fail at the same time, cluster adjustment will be unsuccessful leaving lots of island sensors.

Based on the above analysis we complete the adaptive fault detection and recovery. In the next section, we will discuss and evaluate the effectiveness of our proposed mechanism.

## 5. Simulation and Discussion

### 5.1. Evaluation Indexes

In the simulation process, a statistical stochastic-based method is used to accomplish our experiments. Assume that the probability of the *m*-th class fault of the *i*-th node during a period *L* follows a normal distribution $\left(\mu_{m}(i),\sigma_{m}(i)\right)$. We randomly initialize the expectation of fault probability within a certain range for different on-road sensors and observe the system during a period *L* which consists of many time-slices. In one time-slice *l*, nodes randomly fail according to the assumed expectation. In this way, after observing the system for a certain amount of time slices, we can establish an initial on-road sensor system with a fault statistics function. In this section, we will evaluate the proposed fault tolerance mechanism from three aspects: the effect on the deployment of backup on-road sensors, the deployment of redundant cluster heads and the ADR method, respectively. The detailed evaluation indexes are discussed below:

#### 5.1.1. The Analysis for the Deployment of Backup On-Road Sensors

● The trade-off between cost and fault tolerance of the backup on-road sensor deployment

This index describes the relation between the deployment cost and the number of the backup on-road sensors which is calculated by $E_{s}\times N_{s}$, where the network scale is $N_{s}$ and the deployment expanse is $E_{s}$. In addition, the *N* − *x* principle is considered in this section, in order to keep the cost within an acceptable range with a variety of fault tolerance scenarios.

● The accuracy of the backup on-road sensors deployment

Let $N_{e}(l)$ be the number of backup sensors which is correctly deployed at the physically damaged sensor in the *l*-th simulation, and let $N_{f}\left(l\right)$ be the total number of sensors in the same simulation. As a result, the average mismatch rate $\rho_{s}$ of backup sensor nodes in the *l*-th simulation can be defined as:

(23) $$\rho_{s}=\frac{\sum_{i=1}^{L}1-\frac{N_{e}\left(l\right)}{N_{f}\left(l\right)}}{L}$$

In the following simulation section, we will discuss the influence on the deployment mismatch rate of each parameter in the backup sensors optimization model.

● The improvement on the communication reliability

Let $N_{LC}\left(l\right)$ represent the number of failed on-road sensors which lose communication with the cluster head. Then the average communication successful rate $\rho_{c}$ in the *l*-th simulation can be defined as:

(24) $$\rho_{c}=\frac{\sum_{i=1}^{L}1-\frac{N_{LC}\left(l\right)}{N_{s}}}{L}$$

In the following simulation section, we will compare the communication successful rate before and after the backup on-road sensor deploying when some original on-road sensors are physically damaged.

#### 5.1.2. The Analysis of the Effectiveness of the ADR Protocol

● The improvement of network structure resilience

There will be a set of on-road sensors losing communication with the management center because of a sensor’s physical failure. Let $C_{DB}\left(l\right)$, $C_{RR}\left(l\right)$ and $C_{CR}\left(l\right)$ represent the number of nodes which is recovered by backup sensors, route adjustment or cluster adjustment, respectively. According to the analysis on the average of these three numbers during *L* times simulation, we can verify whether the ADR protocol, which consists of direct recovery by backup on-road sensors, route adjustment and cluster adjustment, is correct and effective enough to deal with the system fault.

#### 5.1.3. The Analysis for the Redundant Cluster Head Deployment

● The contributions to the improvement of QoS

Let $d_{i}$ represent the number of hops from the *i*-th on-road sensor to the nearest cluster sensor. The average number of hops for each node in the system can be given by $d_{av}=\sum_{i=1}^{N}d_{i}/N$ $d_{av}=\sum_{i=1}^{N}d_{i}/N$. According to the different value of $d_{av}$ with different parameters of the optimization model, we can evaluate the improvements of QoS which benefit from the redundant cluster heads deployment.

● The impact on the resilience of network structure

Let $N_{nr}\left(l\right)$ denotes the total number of on-road sensors which are still unable to resume communication after recovery with backup nodes and ADR mechanism at the *l*-th simulation. By considering the average of $N_{nr}\left(l\right)$ in L times simulations we can evaluate the network resilience in the scenario with or without the redundant cluster heads deployment.

### 5.2. Simulation Settings

We will observe the analytical results of this paper based on the Matlab software. Different on-road sensor scenarios are initialized which are composed of N on-road sensors with various failure rates. The scale of the system is defined as $N=N_{u}\cdot N_{oc}$, where $N_{oc}$ represents the initial number of the clusters, and $N_{u}$ is the number of the on-road sensors which belong to each cluster. In a cluster, every on-road sensor can communicate with the management center through the cluster head node. The failure rate denotes the probability of the sensor becoming damaged and it will be used in our objective function to set up the deployment weights of backup sensors. The parameters we considered for failure rate are the natural environment, topological construction, and traffic pattern. Because of the uncertainty of the natural environment, the failure rate is a normal distributed random variable between 0 and 1 with an expectation probability. Considering the influence of topological construction and traffic pattern, on-road sensors closer to a center cluster head will have higher expectation probability of failure rate. The system is firstly observed for a period in order to obtain an initialized scene with historical failure probability statistics. In addition, different sets of the constant coefficients $\lambda_{1}$ and $\lambda_{2}$ in the optimization model are set to evaluate the fault tolerance performance with different fairness between the cost and significance in objective deployment model. We have assumed $\lambda_{1}+\lambda_{2}=1$, if $\lambda_{1}>\lambda_{2}$, the backup sensors’ deployment will put more emphasis on cost savings, as a result, decrease the number of backup sensors deployed. If $\lambda_{1}<\lambda_{2}$, we will deploy as many necessary backup sensors as we can. By setting reasonable values of $\lambda_{1}$ and $\lambda_{2}$, we can achieve a balance between cost and significance to realize a good backup sensor deployment.

The detailed simulation parameters are shown in Table 2. Notice that in our simulation, in order to obviously observe the effect of deployment and recovery, we magnify sensors’ failure probability appropriately compared with the real-world network. Moreover, we use a desktop with 3.6 GHz dual-core processors and 8 GB of RAM to run the proposed algorithm. The corresponding results are averaged over a lot of simulation runs.

### 5.3. Analytical Results and Discussions

#### 5.3.1. Quantitative Analysis for Backup On-Road Sensors

**Figure 12:**
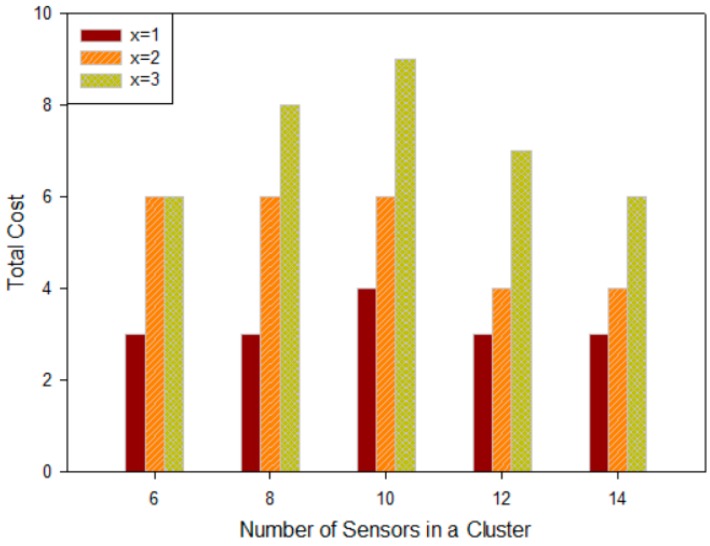
Relationship between number of sensors in a cluster and deployment cost.

In our theory, the deployment of backup on-road sensors should consider the constraint of the *N* − *x* principle. Figure 12 illustrates the relation between *x* in the *N* − *x* principle and the deployment cost of backup nodes, when there are various numbers of on-road sensors in a cluster. The deployment cost can be represented by the number of backup sensors in the system if they possess the same $E_{s}$. This figure implies that the deployment cost of backup nodes increases as the *x* in the *N* − *x* principle increases while the total number of the on-road sensors $N_{u}$ is fixed. This trend is consistent with the conclusion drawn in Figure 4 when x≤μ. While *x* is fixed, the deployment cost of backup nodes firstly increases and then decreases with the increase of the total number of the on-road sensors $N_{u}$. This trend coincides with the characteristic of binomial distribution that represents the fault probability. According to the *N* − *x* principle, in a cluster of $N_{u}$ on-road sensors, when there are *x* failed nodes, the system will not be affected. When $N_{u}$ is small, the number of backup sensors is small because of the small system scale. The number of backup sensors increases with the growth of system scale $N_{u}$, and as a result the cost increases too. However, when $N_{u}$ gets larger and larger, the system scale becomes large enough. It will be easier to meet the *N* − *x* principle so the number of backup sensors becomes less and less and correspondingly as a result the deployment cost is reduced. Therefore, when x is fixed, the cost will first increase and then decrease. Moreover, the larger the x is, the more obvious the trend will be. This implies that we can establish a reasonable *N* − *x* principle in terms of system scale. To simplify the analysis, we use the *N* − 1 principle in the following simulation.

#### 5.3.2. Performance Evaluation on the Proposed Algorithm

**Figure 13:**
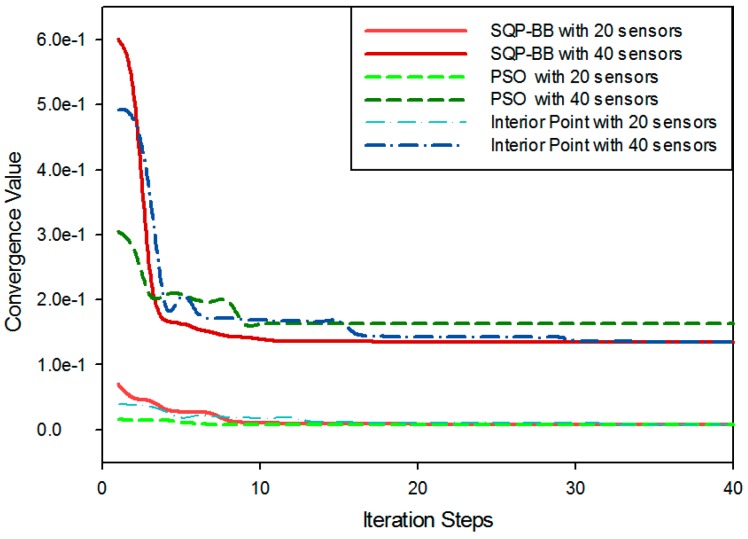
Convergence comparison between the proposed and other algorithms.

Before the results discussion, we firstly verify the performance for the proposed solving algorithm. The scale of the simulation scene is one cluster with 20 or 40 on-road sensors. We present the computational results for solving the proposed deployment problem by SQP with BB used by our paper, Interior Point Method with BB, and Particle Swarm Optimization (PSO) algorithm that represents the kind of intelligent optimization algorithms. The convergence performance results of the three algorithms are compared, as shown in Figure 13.

By comparing the red line and the blue line, we can see that SQP has a greater convergence rate than the interior point method for solving nonlinear programming problems. The green line represents the intelligent optimization algorithm for which we used the PSO algorithm, which uses a number of agents (particles) that constitute a swarm moving around in the search space looking for the best solution. PSO is an intelligent algorithm with a fast convergence rate but it is generally used to deal with unconstrained mixed integer programming problems, so for our problem, we need to add a penalty function to convert the constrained nonlinear programming problem into an unconstrained one and to escape from local optimal solutions, but the design of the penalty function is very complicated so it is difficult to get a precise result with an improper penalty function. As shown in the figure, the instability of PSO is obvious where it cannot converge to the global optimal solution as the red and blue lines. With the increase of the sample space scale, the instability is becoming more and more obvious. According to the simulation results of Figure 13, we can conclude that the SQP combined with the Branch and Bound algorithm is a reasonable and effective method to solve our objective function, which is essentially a constrained nonlinear 0–1 programming problem with quadratic terms.

#### 5.3.3. Deployment Accuracy Rate Analysis for Backup On-Road Sensors

**Figure 14:**
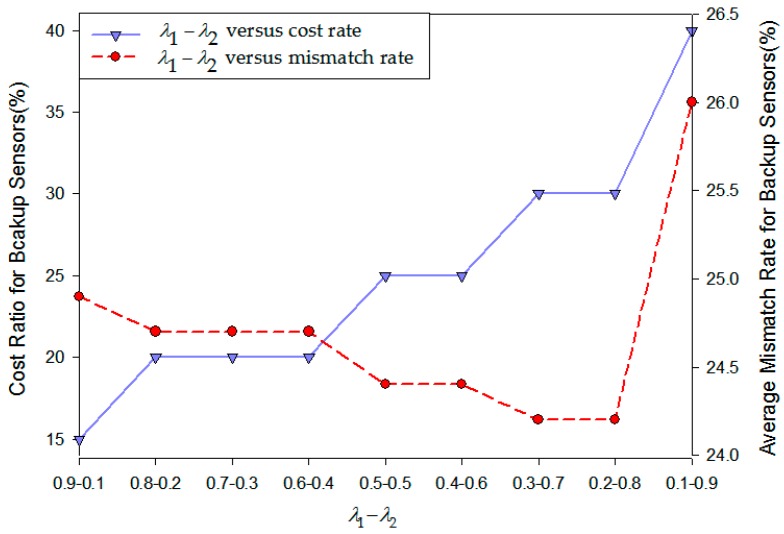
Backup sensor deployment cost ratio and average mismatch rate with different values of $\lambda_{1}$, $\lambda_{2}$.

Consider a scene within one cluster which is composed of 20 on-road sensors and every sensor has a distinctive fault probability. Firstly, by using the backup sensors deployment model which is shown in Equation (13), we can calculate $N_{s}$ (the number of backup sensor nodes in this cluster) and $SP^{*}$ (an array composed of (0, 1) binary variables which represents whether there is a backup node on the *i*-th original on-road sensor). There are two factors we should consider to evaluate the accuracy of the backup sensor deployment. The first factor is the cost ratio of backup sensor deployment that can be computed by $N_{s}/N$ (where N is the total number of sensors in a cluster). The second factor is the average mismatch rate of backup sensor deployment. By using various fault probabilities for each sensor we directly randomize 100 sets of data and each set of data is an array of (0, 1) binary variables which represents the positions of backup sensors. We then compare this array with the vector SP and add up the numbers of mismatched sensors for 100 simulation runs. Its average will be the mismatch rate of backup on-road sensor deployment. It should be noticed that mismatched sensors include two types: one is the actually failed sensors without backup ones deployed near them and the other is the work-normal sensors with deployed backup ones. As a result, the comparative result for the cost ratio and the average mismatch rate of backup sensors on the conditions of different $\lambda_{1}$, $\lambda_{2}$ is shown in Figure 14.

From problem (13) we can know that when *λ*_1_ is larger, the optimization goal prefers to focus on minimizing the cost of backup sensor deployment; when *λ*_2_ is larger, the optimization goal prefers to focus on recovering system failure probability. As shown in Figure 14 by the purple-solid line, the cost ratio of backup sensor deployment grows steadily with the decrease of *λ*_1_. Correspondingly, in the red-dashed line, the average mismatch rate of backup sensors deployment firstly decreases and then increases with the growth of *λ*_2_. This is because when *λ*_2_ is smaller, the model places more attention on cost savings. Consequently, the number of backup on-road sensors is less than the actual need so that the mismatch rate is high. When *λ*_2_ is larger, the model places more attention on compensating the fault probability for each sensor. As a result of the waste of resources, deploying too many backup sensors in the system leads to an increment of the mismatch rate. Based on the above analysis, we propose to use intermediate values of *λ*_1_ and *λ*_2_. This way, we can not only guarantee the deployment cost within a reasonable range but also keep mismatch rate at a low level.

**Figure 15:**
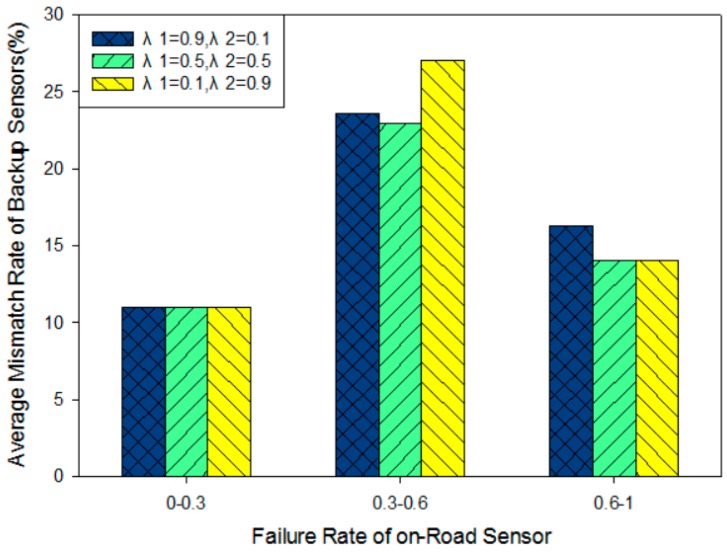
Relation between average mismatch rate and failure rate for each sensor with different values of $\lambda_{1}$, $\lambda_{2}$.

Figure 15 explicitly depicts the relation between the average mismatch rate of backup sensor deployment and failure probability for each on-road sensor. The fault probability of the node is divided into three classes [0–0.3], [0.3–0.6] and [0.6–1] from low risk to high risk in light of the threshold $\varphi_{L}$, $\varphi_{H}$. We can respectively evaluate them by a statistical method.

Figure 15 illustrates that the accuracy rate for backup sensor deployment is higher when the failure rate is in [0.6–1] and [0–0.3]. However, when the failure rate is in the range of [0.3–0.6], the deployment work has low accuracy. The model with *λ*_1_ = 0.9 and *λ*_2_ = 0.1 is more focused on considering minimizing deployment cost, and the number of backup on-road sensors will be less than the real failed nodes in a practical system. Consequently, for sensors with higher fault probability in [0.3, 0.6] and [0.6, 1], the mismatch rate is much higher. Relatively the model with *λ*_1_ = 0.1 and *λ*_2_ = 0.9 is more focused on considering the fault probability of every node. The backup sensor deployment priority will be ranked from high to low fault probability. In this case, we can successfully deploy backup sensors just at the point where the original on-road sensors have high failure probability, but in the intermediate fault probability [0.3, 0.6], the number of backup sensors could be more than the number of actually failed sensors which leads to a high mismatch rate in the deployment work.

Combining the above analysis, intermediary values of *λ*_1_ and *λ*_2_ can make our model achieve both aspects of the balance of cost and fault probability. For on-road sensors with higher failure probability, the deployment method basically guarantees the coverage of the backup nodes and it could be not too sensitive to cost. For the ones with lower failure probability, the model ensures that there will not be waste in the backup sensor deployment. The number of backup on-road sensors is extremely close to the number of actually failed sensors and hence the mismatch rate performance is the lowest for each case. Consequently, when *λ*_1_ = 0.5 and *λ*_2_ = 0.5 in the model, the system can achieve a good balance between reliability and cost. This is consistent with the conclusion in Figure 14.

#### 5.3.4. Communication Reliability Improved by Backup On-Road Sensors Deployment

**Figure 16:**
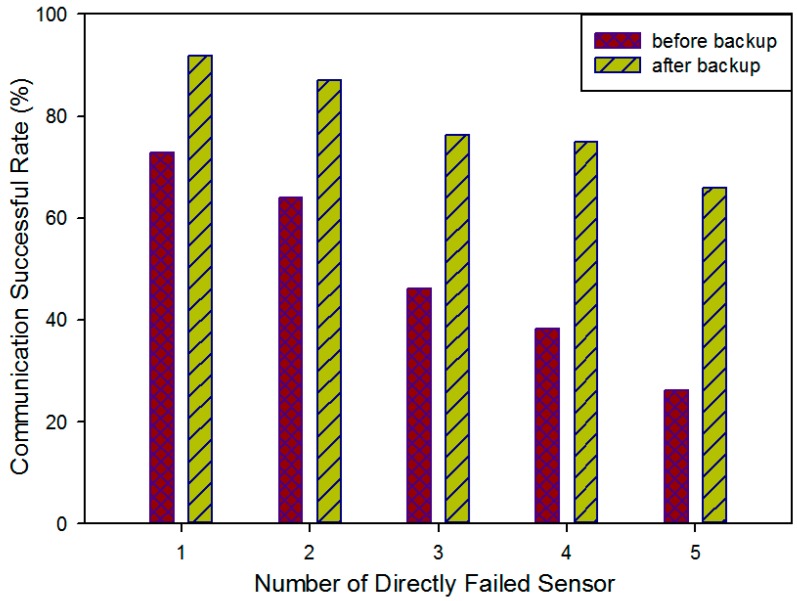
Communication successful rate before and after backup on-road sensor deployment.

In this section, we run the simulation in a cluster with 26 on-road sensors with various fault probabilities. We randomly generate five kinds of scenario with different numbers of physically failed sensors 1, 2, 3, 4 and 5. We use the network communication successful rate defined in Equation (24) to evaluate the communication reliability improved by the backup on-road sensors deployment model. The analytical results are given in Figure 16.

As shown in this figure, the purple columns represent the network communication success rate when there are a couple of physically failed sensors, the yellow columns represent the network communication success rate after recovery with deployed backup sensors. It can be seen that the awakening backup sensors can effectively increase the communication success rate and as a result restore the system’s normal communication. Our method can basically address the communication reliability problem by awakening backup sensors in the corresponding position, however, limited by the deployment cost, the on-road sensor’s backup work cannot recover all the failed communication sensors, especially when the number of physically damaged sensors is large. Hence with the increase of the number of physically failed sensors in the system, the network communication success rate decreases whether before or after backup sensor recovery. As a result, more physically failed sensors in a system will reduce the recovery effect of backup sensors, so it will be necessary to recover effective system communication with route adjustment and cluster adjustment.

#### 5.3.5. Resilience of Network Structure

**Figure 17:**
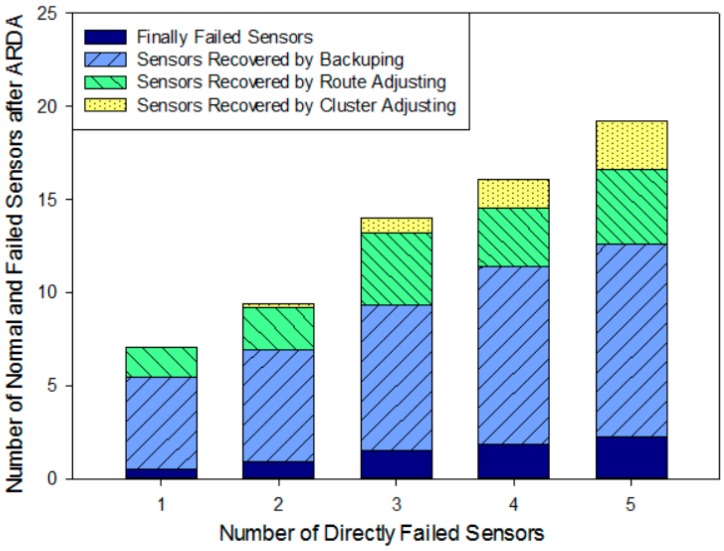
Number of normal and failed sensors after applying ARDA versus the number of physically failed sensors.

For the same scenarios as the previous section, we add up simulation results 100 times and their average can be used to evaluate the resilience of the network structure. When a failed sensor has a backup node deployed on it, it will be recovered by the backup node directly. Otherwise, if there is no backup node deployed on it, some other on-road sensors will lose communication with the management center because of this physically failed sensor. We call this physically failed sensor a “directly failed sensor” and other failed sensors that lose communication are “indirectly failed sensors”. When there is a trouble in the system, the failed on-road sensors include both directly and indirectly failed sensors. Every column in Figure 17 represents the total number of failed on-road sensors in a cluster where the *x*-coordinate indicates how many directly failed sensors there are actually. The ADR protocol is executed to recover the network, including the route adjustment and cluster adjustment, but if the sensor cannot be recovered by cluster adjustment or the restriction on the hops cannot satisfy the QoS requirement, these sensors will become the island sensors. Ultimately, physically failed sensors and island nodes cannot be recovered by the ADR mechanism.

The network recovery ability is shown in Figure 17. Firstly, the average number of failed sensors increases with the increase of the number of directly failed sensors. Without any hop restriction on QoS, almost all the failed sensors can be recovered by backup sensor nodes, route adjustment and cluster adjustment. When the number of physically failed on-road sensors are 1, 2, 3, 4 and 5, the proposed fault-tolerance mechanism can recover 92.8%, 90.4%, 89.2%, 88.6% and 88.3% of the failed on-road sensors, respectively. The average recovery rate reaches 89.9%, which ensures a good fault-tolerance performance for different failure cases. It is also shown that the failed sensors recovered by direct backup account for the largest proportion of the total recovered sensors, and route adjustment manner gives the second largest amount. In particular, when there is only one directly failed sensor in the cluster, the failed sensors can be completely recovered by backup on-road sensors and route adjustment. With the increase in the number of directly failed nodes, the proportion of cluster adjustment gradually increases, while at the same time, the number of remaining failed nodes after the ADR protocol adjustment increases too. These nodes are comprised of directly failed sensors and island sensors.

Based on the above analysis, the ADR mechanism can dynamically and effectively recover the system faults. Especially when there are a few physically failed sensors in a cluster, the number of on-road sensors remaining failed after using ADR are less than the actually physically failed sensors. Furthermore, the low proportion of the cluster adjustment guarantees the stability of the network.

#### 5.3.6. The Contribution of the Deployment with Redundant Cluster Heads to the Improvement in QoS

This section elaborates the original simulation scenario with six clusters and each cluster has 20 on-road sensors. With the fixed $R_{k}$ which is the coverage radius of the *k*-th cluster, we can figure out an array composed of (0, 1) binary variable named CP* which represents whether there is a redundant cluster head in the *i*-th on-road sensor. In a cluster, we add up hops of each sensor to the cluster head and calculate its average, which is named as $d_{av}$ which is the average hops of the cluster. $d_{av}$ is used to evaluate the contribution of the redundant cluster head to the improvement in the quality of service. Figure 16 shows the performance of the redundant cluster head deployment with different values of *λ*_1_, *λ*_2_ and different coverage constraint conditions.

**Figure 18:**
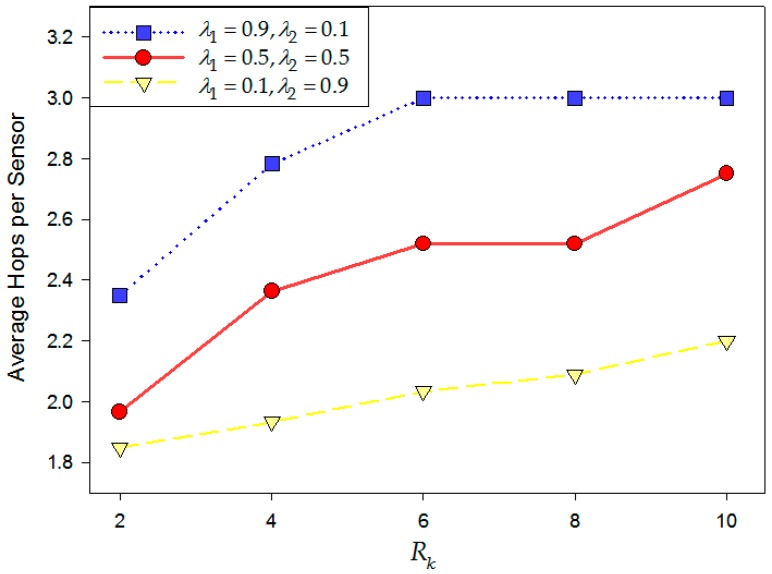
Average hops of each sensor versus *R_k_*.

From Figure 18, it can be seen that when *λ*_1_ = 0.1 and *λ*_2_ = 0.9, the deployment model of redundant nodes focuses on the improvement of network hops, so the trend of the yellow-dashed line is steadily going downwards with the decrease of $R_{k}$. We can see the average hops per on-road sensors are not sensitive to coverage constraints. The reason is that spending much more on deployment can make the on-road sensor system become denser. For the case of *λ*_1_ = 0.9 and *λ*_2_ = 0.1, the model prefers to focus on the deployment cost. If $R_{k}$ is large, all of the backup sensors will be deployed on the existing cluster heads which has a low deployment cost $E_{L}$. This way, the redundant cluster head deployment realizes a full backup and the average hops of the system would not decrease until we have enough sensor nodes in the system. As a result, the average hops decrease approximately linearly with the decrease of coverage constraint $R_{k}$, from 6 to 2, as shown by the blue-dotted line. If *λ*_1_ = 0.5 and *λ*_2_ = 0.5, the deployment strategy focuses on the balance between improvement in quality of service and deployment cost. As shown in the red-solid one, when $R_{k}$ is large, a limited number of redundant cluster heads will be preferentially deployed to reduce the system average hops. However, with the decrease of $R_{k}$, the system prefers to deploy these redundant cluster heads on the original cluster heads first so as to reduce deployment costs. When there are enough redundant cluster heads left in the system, they can be deployed on the other sensor nodes to form more clusters. By this way, the improvement of system average hops is superior to what is indicated by the blue-dotted line.

#### 5.3.7. The Influence of Redundant Cluster Heads Deployment on the Network Resilience

The simulation scenario in this section is composed of six clusters and each cluster has 26 on-road sensors. On the condition of *λ*_1_ = 0.5 and *λ*_2_ = 0.5, the deployment strategy takes both backup on-road sensors and redundant cluster heads into account at the same time to realize a fault-tolerant on-road sensor system. According to the various fault probabilities of each sensor, we randomly generate five kinds of cases in which the number of physically failed sensors is 1, 2, 3, 4, and 5, respectively. We add up the number of failed sensor nodes after the ADR adjustment in each simulation for 100 times and its average can be used to evaluate the network resilience when there is redundant cluster head deployment in the system or not. Remaining failed sensors the in y-coordinate consist of island nodes and directly failed sensors which cannot be recovered by backup sensors. The island nodes include ones that cannot be recovered by cluster adjustment and ones that do not satisfy the hop limits (the hop limit is assumed to be 8 or 10 in our simulation).

**Figure 19:**
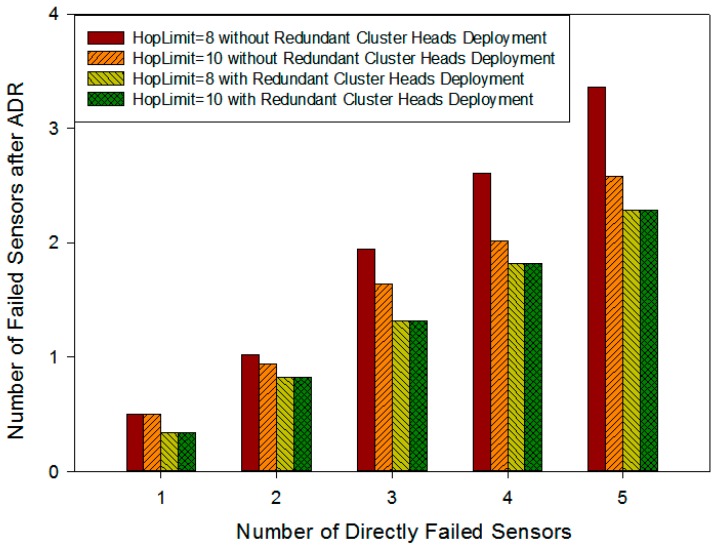
Number of failed sensors after ADR versus number of directly failed sensors.

Firstly, Figure 19 shows that in the scenario without redundant cluster head deployment, if there are more physically failed sensors, there will be more sensors losing communication with the management center in the system, while at the same time, the more stringent the hops constraint is, the more remaining failed sensors are left after ADR adjustment. This is because with the increase of the number of failed sensors, cluster adjustments become more and more frequent. The strict hops limit places restrictions on the sensors’ communication, which makes them unrecoverable by cluster adjustment and this causes more island nodes.

Correspondingly, it can be seen that the deployment with redundant cluster heads effectively narrows down the size of each cluster so as to reduce the impact of the hop limit on cluster adjustment and the number of island nodes. Moreover, the number of remaining failed sensors after ADR adjustment is reduced considerably. This shows that the total number of failed sensors after ADR adjustment with deployment of cluster redundant nodes is lower by 35.7% at most than without their deployment when the number of physically failed sensor is 5 and the communication hops limit is 8. In addition, the scale of the system with redundant cluster heads deployment must be smaller than the original system without redundant cluster heads deployment, so in the same scenario, the hop limit would not affect cluster adjustment any more. In other words, there are no island nodes which cannot be recovered by cluster adjustment because of the hop limit. As a result, in Figure 19 whether the hop limit is 8 or 10, the average number of failed sensors after ADR is always same in the redundant cluster head deployment situation.

## 6. Conclusions

On-road sensor networks place increasing demands on critical communication with the rapid development of ITS applications, such as driving safety and automatic driving. To satisfy these requirements, this paper probes into the problem facing the fault tolerance in OSNRs. According to the characteristics and topology of an OSNR, a complete and dedicated fault tolerant architecture is studied, which consists of four phases: planning, deployment, recovery and evaluation. In the planning and deployment phases, we firstly introduce a two-objective optimization model of backup on-road sensor deployment with an *N* − *x* principle constraint condition, which allows a trade-off between cost and fault recovery. Through determining the position where the backup sensors are placed, the communication reliability can be basically guaranteed. Then we present a redundant cluster head deployment model in order to improve the sensors’ communication hops under a coverage-constrained cost. From this model, we can form a dense cluster-based OSNR as much as possible within some cost constraints so that the network resilience can be improved. After converting these two-objective optimizations into single ones, a joint BB and SQP algorithm is proposed to solve them with a good convergence performance. However, limited by the cost constraint, on-road sensors backup work cannot recover all the failed communication sensors, especially when the number of physically damaged sensors is large. Therefore in the recovery phase, we design an ADR protocol to recover from on-road sensor faults by routing and cluster adjustment. The sensors which still lose the communication after awaking the backup sensors can be adaptively detected and the corresponding communication routing can also be recovered effectively by the ADR protocol. We discuss any possible failure situation and propose a routing and cluster adjustment strategy. Finally the quantity-based evaluation results are shown from the perspectives of communication reliability and network resilience. As known from the results, our proposed on-road sensor backup method can basically guarantee a node’s successful communication rate and combined with ADR, the total fault tolerance can achieve a 90% fault recovery rate when the number of directly failed sensors is 5. Besides, the redundant cluster head deployment can further reduce the average number of failed sensors by 35.7% if the sensors’ communication hop limit is 8, which provides an effective contribution to the network resilience.

There are still some tasks that are needed to be accomplished in the future. Firstly, when considering the communication reliability, the evaluation factor is determined by whether the road-side sensors can communicate normally with the cluster heads through the hop-by-hop transmission manner. However, if we focus on some specific technologies like Zigbee and WiFi, there are some other indexes that can be considered, such as the probabilistic pack loss [24,25,26] and coverage [27,28,29] performance. Moreover, due to this limit, this paper only gives an analytical simulation result. Some simulators like NS and OPNET are well-established and have been applied for these specific WSN communication technologies [30,31,32,33], such as Zigbee, WiFi, LTE and so on. By choosing a WSN communication technology, we can extend the simulation work on these well-established simulators to verify more indicators.

## Figures and Tables

**Table 1:** Notations used in this paper.

Notation	Description
*N*	Total number of the on-road sensors
$N_{s}$	Total number of the backup on-road sensors
$N_{c}$	Total number of the redundant cluster heads
*SP*	A binary vector of position for the on-road sensors
*CP*	A binary vector of position for the redundant cluster heads
$E_{s}$	Expense of purchase and installation per backup on-road sensor
$E_{c}$	Expense of purchase and installation per cluster head
*RR*	Reservation ratio for scalability
$C_{k}$	The *k*-th cluster of the system
$R_{k}$	Coverage radius of the the *k*-th cluster
*M*	Number of the fault types
$F_{ij}$	Frequency of the *j*-th type fault of the *i*-th node

**Table 2:** Simulation parameters.

Parameter	Definition	Value
x	*x* Nodes fail in *N* nodes according to the *N* − *x* principle	{1,2,3}
$N_{oc}$	The initial number of the clusters	6
$N_{u}$	The number of on-road sensors in a cluster	{20,26}
$p_{i}$	The fault probability of the *i*-th on-road sensor	0.5
$\varphi_{L}$	Low threshold of fault frequency	0.3
$\varphi_{H}$	High threshold of fault frequency	0.6
$R_{k}$	Coverage radius of the *k*-th cluster	{2,3,...,10}
$E_{s}$	Procurement and deployment expanse for a single sensor	1 unit
*RR*	Reservation Ratio for scalability	0.2
$E_{L}$	Deployment cost of a reuse of the existed cluster head	1 unit
$E_{H}$	Deployment cost of a new cellular node’s machine	7 units
